# Grain Refinement and Microstructural Evolution in Cobalt-Saving 18Ni (300) Maraging Steel via Cold Deformation-Cyclic Solution Treatment

**DOI:** 10.3390/ma18132947

**Published:** 2025-06-21

**Authors:** Feng Huang, Zhe Cheng, Defa Li, Wei Zhang, Zhili Hu

**Affiliations:** 1 Hubei Key Laboratory of Advanced Technology for Automotive Components, Wuhan University of Technology, Wuhan 430070, China; huangfeng@whut.edu.cn (F.H.); 15802739678@163.com (Z.C.); 2 Hubei Longzhong Laboratory, Xiangyang 441022, China; 3 School of Mechanical and Vehicle Engineering, West Anhui University, Lu’an 237012, China; 04000144@wxc.edu.cn

**Keywords:** maraging steel, cold deformation, cyclic solution treatment, strengthening and toughening mechanism

## Abstract

To solve the problem of inadequate plasticity of traditional processing routes in improving the plasticity of novel Co-saving 18Ni (300) maraging steel, a cold deformation-cycle solution treatment process was developed. Through systematic characterization and tensile property testing, the study focuses on elucidating the impact of the number of solution treatments on the microstructure and mechanical behavior. The results showed that with a 30% cold deformation, three times of solution treatment at 860 °C for 10 min refined the original austenite grains (equivalent circle radius: 3.3 μm) and martensite structure (length and width: 7 μm and 1.3 μm, respectively) to the utmost extent. The grains became uniformly equiaxed, and the texture was eliminated, and a moderate content (4.5%) of retained austenite was formed. At this time, the material achieves the best match between strength (tensile strength of 1240 MPa) and plasticity (elongation of 9.93%), which are increased by 15.3% and 94.3%, respectively, compared with the traditional process. Mechanistic analysis revealed that grain refinement and uniform equiaxialization were the primary drivers for enhancing strength and plasticity. This study has demonstrated that the cold deformation-cyclic solution treatment process is an effective methodology for tailoring the microstructure and mechanical properties of maraging steel.

## 1. Introduction

Maraging steel, a novel ultra-high-strength steel, exhibits an exceptionally low carbon content (typically <0.01%) compared to conventional steel. This characteristic distinguishes its strengthening mechanism from traditional steels: rather than relying on carbides, it achieves reinforcement through precipitates [1,2,3]. Nickel (Ni) serves as the primary constituent of these precipitates. During aging, Ni precipitates from the matrix and combines with titanium (Ti), molybdenum (Mo), and aluminum (Al) to form numerous fine strengthening phases (e.g., η-Ni_3_Ti, Ni_3_Mo, and β-NiAl). These phases act as barriers to impede dislocation motion, thereby enhancing the matrix strength via an obstacle-hardening mechanism.

The commercially prevalent maraging steel is the cobalt (Co)-containing 18Ni grade. Cobalt addition not only enhances the steel’s strength but also improves its plasticity. This dual benefit arises from two mechanisms: first, increasing Co content elevates the martensite start temperature (Ms), ensuring complete austenite-to-martensite transformation during cooling; second, Co reduces the solid solubility of alloying elements, retards substructure recovery in martensite, and promotes denser distribution of precipitated phases. Additionally, Co effectively refines grain size [4,5]. However, due to Co’s scarcity and high cost, efforts have shifted toward developing Co-free or Co-saving maraging steels, with performance improvements achieved through process optimization.

Current research on Co-saving 18Ni maraging steel predominantly focuses on optimizing heat treatment processes to achieve enhanced strength-plasticity coordination, with a particular emphasis on improving plasticity. Grain refinement remains the most effective strategy to simultaneously enhance both the strength and plasticity of metallic materials. Studies have shown that when the grain size is refined to a significant extent, the strength and plasticity of the material tend to reach a plateau [6]. Therefore, achieving an optimal grain size through precise process control is essential for further improving the mechanical properties of maraging steel, as it balances the synergistic effects of strengthening mechanisms and plastic deformation capacity.

Grain refinement in metallic materials can be achieved through various approaches, including alloying element addition, cold deformation, and heat treatment [7,8,9]. Cold deformation processing disrupts the original austenite grains [10], effectively refining the martensitic microstructure via strain-induced fragmentation [11]. However, in traditional maraging steels, a mere increase in cold deformation degree often leads to diminishing returns in grain refinement and may induce abnormal microstructures (e.g., uneven deformation or crystalline texture), thereby deteriorating mechanical properties. Cyclic solution treatment has been demonstrated to enable further grain refinement and optimize microstructural morphology [12]. Notably, existing studies have predominantly focused on maraging steels without prior cold deformation [13,14,15], leaving a critical research gap regarding the synergistic effect of cold deformation and cyclic solution treatment on grain refinement and microstructural homogeneity.

Therefore, this study focuses on Co-saving 18Ni steel and addresses the microstructural defects induced by cold deformation by integrating cold deformation with cyclic solution treatment. Building on the author’s previous research [16], this work emphasizes experimental investigations into the effect of the number of solution treatments. The objectives are to: (1) elucidate how cyclic solution treatment influences cold-deformed microstructures, (2) decipher the microstructural evolution mechanisms and strength-ductility synergistic principles during the cold deformation-cyclic solution treatment process, and (3) establish ideal microstructural and mechanical property foundations for subsequent aging treatment while enabling potential for further Co content reduction in maraging steel.

## 2. Material and Methods

### 2.1. Materials and Processing

In this experiment, the original cast billet of Co-saving 18Ni (300) maraging steel from a certain factory was used as the test material. The main chemical compositions are shown in Table 1. Approximately 15 kg of the billet was forged and then cut into several sheets with dimensions of 200 mm × 50 mm × 5 mm to facilitate cold deformation and cyclic solution treatment tests. All specimens were sanded with sandpaper before being heat-treated.

**Figure 1:**
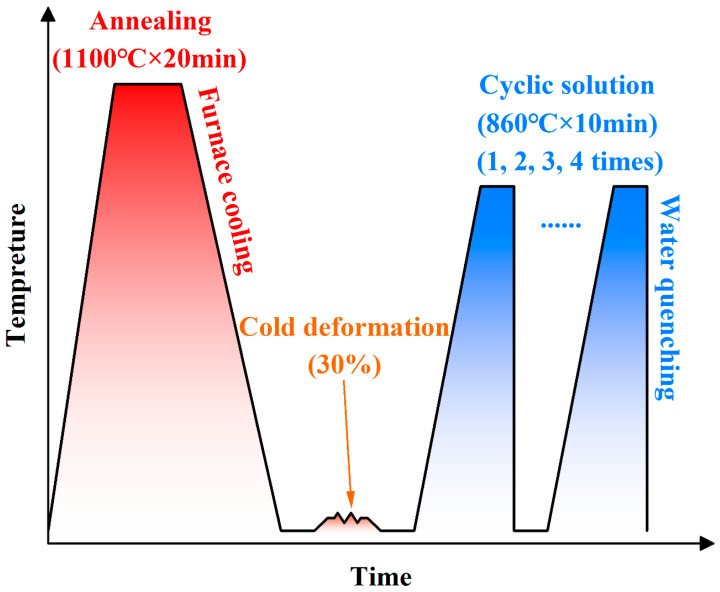
Process Flow Diagram.

According to the process flow diagram in Figure 1, all steel plates first underwent a vacuum annealing pretreatment of 1100 °C for 20 min, followed by furnace cooling to room temperature, to eliminate residual stresses in the material after forging. The equipment utilized was a vacuum atmosphere furnace with an operating voltage of 380 V, rated power of 12 kW, maximum temperature of 1550 °C, and argon (purity: 99.9%) as the protective gas. Heating was achieved via resistance wire induction at a rate of approximately 5 °C/min. Then, cold deformation was conducted using an N-100T variable-cross-section cold rolling mill (Huangshi Huali Metalforming Machine Tool Co., LTD, Huangshi, China) with a nominal force of 1000 kN. The steel plates were repeatedly rolled to achieve the preset deformation of 30% (from 5 mm to 3.5 mm). Finally, cyclic solution treatment was performed using a box-type resistance furnace with an operating voltage of 220 V, a rated power of 4 kW, and a maximum temperature of 1000 °C. The furnace employed resistance wire induction heating at a rate of approximately 15 °C/min. Before heat treatment, the air inside the furnace is evacuated to 10 Pa, then argon is backfilled to standard atmospheric pressure, and this process is repeated 2–3 times. During the heat treatment process, the gas pressure inside both furnaces is standard atmospheric pressure (approximately 1.01 × 10^5^ Pa). Once the set temperature was reached, the specimens were held isothermally for a specified duration and then water-cooled to room temperature. The initial temperature of the cooling water is room temperature (approximately 20 °C). The cooling rate was estimated to be approximately 140 °C/s (it took approximately 6 s to cool from 860 °C to 20 °C). The setting of relevant heat treatment process parameters was based on the previous work [16].

The experimental steels subjected to 1–4 times of solution treatment after cold deformation were designated as specimens 1#–4#, respectively. Specimen 0# served as a comparative specimen, prepared via the traditional process (without cold deformation and with only one time solution treatment). Prior to solution treatment, the specimens for 0# and 1# were labeled as 0#-Before ST and 1#-Before ST, respectively.

### 2.2. Methods

Microstructural characterization and mechanical property testing were conducted on specimens in different states.

The microstructure of the materials was examined using a Zeiss Ultra Plus field emission scanning electron microscope (SEM) (Carl Zeiss AG, Oberkochen, Germany) equipped with an energy dispersive spectrometer (EDS) for elemental composition and content analysis. Specimens, sized at 10 mm × 10 mm × 5 mm, were ground, polished, etched with a 4% nitric acid-ethanol solution, and thoroughly cleaned with alcohol before SEM. During SEM analysis, an accelerating voltage of 20 kV was applied, and the back scattered electron mode (BSE) was chosen. Moreover, the secondary electron mode (SE) was employed to observe the morphology of the fracture surfaces of the tensile-tested specimens. Grain size was measured using Image J (Java 8) software.

An Empyrean X-ray diffractometer (XRD) was utilized to characterize the phases of the specimens. The specimen preparation for XRD was identical to that for SEM. The XRD system employed Cu Kα radiation, with a 2θ scanning range of 30–90° and a scanning rate of 10°/min. Martensite and austenite were distinguished based on the diffraction peak angles corresponding to their respective crystallographic orientations, followed by statistical analysis of peak intensities. The XRD data were processed and analyzed using Origin 2022 software.

**Figure 2:**
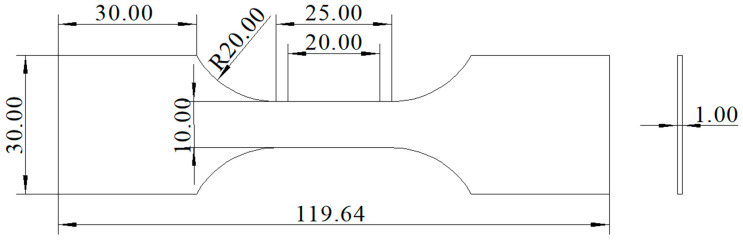
Schematic of the tensile specimen size (all measurements are in mm).

Room-temperature tensile properties of the specimens were evaluated using a Z050 electronic universal material testing machine (ZwickRoell, Ulm, Germany). The equipment had a maximum tensile load capacity of 50 kN and a tensile speed of 0.5 mm/min. As shown in Figure 2, the test specimens featured a parallel section with dimensions of 25 mm × 10 mm × 1 mm and a gauge length of 20 mm. Prior to testing, three 1 mm thick tensile test specimens were cut from a 3.5 mm thick plate. The surfaces and sides of the tensile specimens were ground to prevent stress concentration caused by cutting marks during the test. Three specimens were tested per experimental group to ensure the reliability of the mechanical property data.

## 3. Results and Discussion

### 3.1. Microstructure and Composition Distribution

#### 3.1.1. Microstructural Morphology

**Figure 3:**
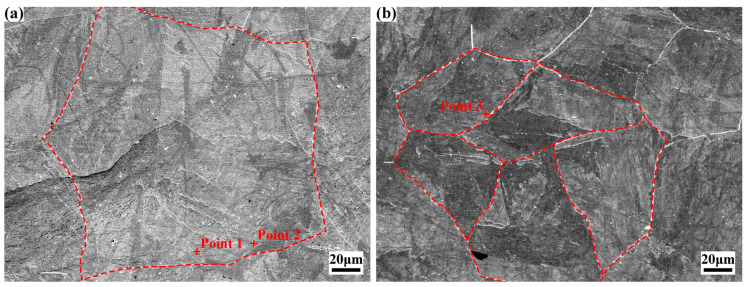
SEM images of the experimental steel before solution treatment: (**a**) 0#-Before ST; (**b**) 1#-Before ST.

Figure 3 shows the microstructural morphologies of specimens 0#-Before ST and 1#-Before ST. Before solution treatment, the microstructure exhibits typical lath martensite with random orientation, and the original austenite grain boundaries are distinctly observable [17]. Martensite within the original austenite does not extend outward, with each martensite block parallel to one facet of the original austenite grain. Cold deformation is demonstrated to significantly modify the material’s microstructural morphology, resulting in reduced sizes of both original austenite grains and martensite.

Figure 3a illustrates specimen 0#-Before ST, which exhibits equiaxed original austenite grains with an axial length of approximately 188 μm. Figure 3b shows specimen 1#-Before ST subjected to 30% deformation, where multiple small austenite grains formed by the fragmentation of a single large grain are observed. These small grains display irregular morphologies, elongated along the rolling direction (73–125 μm in length) and correspondingly reduced in width (approximately 46–55 μm). Both specimens contain uniformly distributed tiny white particles with a diameter of 1–2 μm, with fewer particles present in 1#-Before ST. These particles are likely residual austenite formed during annealing.

The sizes of martensite and original austenite grains were quantitatively statistically analyzed using Image J software. Martensite length and width decreased from 76.1 μm to 53.9 μm and from 6.6 μm to 5.4 μm, respectively. The average area of original austenite grains reduced from 30,769 μm^2^ to 8960 μm^2^, with the corresponding equivalent circle radius decreasing from 99 μm to 53.4 μm.

**Figure 4:**
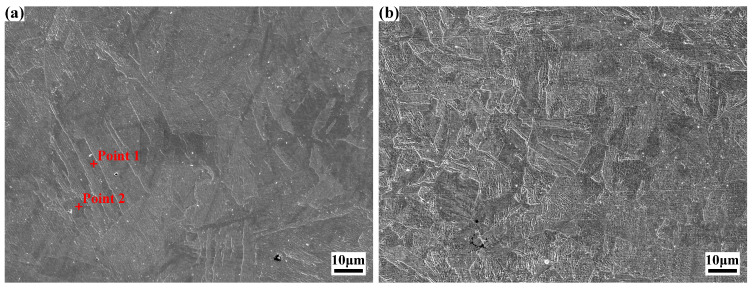
SEM images of the experimental steel after one cycle of solution treatment: (**a**) 0#; (**b**) 1#.

Figure 4 illustrates the microstructural morphologies of specimens 0# and 1# of the experimental steel. After one time of solution treatment, the morphology of lath martensite is highly distinct with random orientation, while the original austenite grain boundaries exhibit blurring. Martensite dimensions were quantitatively analyzed using Image J software. Comparative analysis between specimens 0# and 1# revealed a reduction in martensite length from 47.0 μm to 18.3 μm and a corresponding decrease in martensite width from 4.2 μm to 2.8 μm. The average area of original austenite grains decreased significantly from 27,762 μm^2^ to 317 μm^2^, with the equivalent circle radius correspondingly reducing from 94 μm to 10 μm.

Notably, solution treatment further promoted grain refinement, with a more pronounced effect observed when solution treatment followed cold deformation. Additionally, significant changes in grain morphology were evident: most original austenite grains evolved into equiaxed morphology, which appears to suggest a recrystallization phenomenon. Trace amounts of fine white particles (≈1 μm in size) were dispersedly distributed in both specimens, likely representing residual austenite formed during annealing and solution treatment.

**Figure 5:**
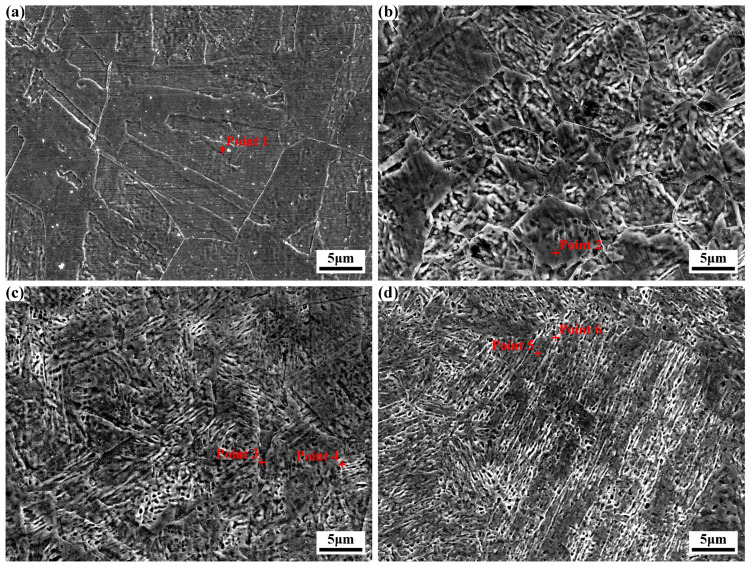
SEM images of the experimental steel after different times of solution treatment: (**a**) 1#; (**b**) 2#; (**c**) 3#; (**d**) 4#.

Figure 5 displays the microstructural morphologies of specimens subjected to different times of solution treatments (Figure 5a is a partial magnification of Figure 4b). Distinct lath martensite structures and original austenite grains are observable. Notably, the martensite size decreases significantly with increasing solution treatment times, though a slight increase is evident after the fourth treatment. Image J software-based statistical analysis reveals that martensite length gradually decreases to 7.0 μm in specimen 3#, then increases slightly to 8.6 μm in specimen 4#, with corresponding width reduction to 1.3 μm. Specifically, martensite lengths in specimens 2#, 3#, and 4# are reduced by 26.8%, 61.7%, and 53.0% relative to specimen 1#, with widths decreasing by 46.4%, 53.6%, and 53.6%, respectively. Significantly, after four times of solution treatment, martensite length exhibits a minor increase rather than continuous reduction, while width remains unchanged.

In Figure 5a, the original austenite grains are relatively large, whereas those in Figure 5b show uneven sizes. By contrast, the original austenite grains in Figure 5c,d are smaller and more uniform. Image J software-based statistical analysis of original austenite grain sizes in the experimental steel subjected to 1, 2, 3, and 4 times of solution treatment revealed average grain areas of 317 μm^2^, 70 μm^2^, 34 μm^2^, and 39 μm^2^, with corresponding equivalent circle radii of 10.0 μm, 4.7 μm, 3.3 μm, and 3.5 μm, respectively. Compared to the specimen after 1 time of solution treatment, the martensite grain sizes of specimens after 2, 3, and 4 times of solution treatment were reduced by 53.0%, 67.0%, and 65.0%, respectively. It can be seen that with the increase in the number of solution treatments, the size of the original austenite grains first decreases and then slightly grows, which is consistent with the change rule of martensite size, indicating that grain refinement has an effect on refining the martensite structure. Meanwhile, some fine white granular structures with a size of about 1 μm can be observed, which may be retained austenite, and with the increase in the number of solution treatments, their volume fraction slightly increases.

#### 3.1.2. Microzone Composition

During the microscopic characterization described above, EDS point analysis was conducted on characteristic regions, with results tabulated in Table 2. For specimens before solution treatment and after one time of solution treatment, elemental distribution within the matrix remained relatively uniform, demonstrating that cold deformation and one time of solution treatment exerted negligible influence on the matrix elemental composition.

For specimens with varying times of solution treatments, the Ni and Co contents in the martensite matrix of specimens 1# and 2# were slightly higher than those in specimens 3# and 4#, though the overall elemental ratios remained similar. In specimens 3# and 4#, discrepancies in Ni and Mo contents were observed between the matrix and white granular structures. The Ni content in white granules (Points 4 and 6 in Figure 5) was marginally higher than that in the matrix (Points 3 and 5 in Figure 5), while other elements exhibited negligible content variations.

#### 3.1.3. Phase Composition and Crystallographic Features

**Figure 6:**
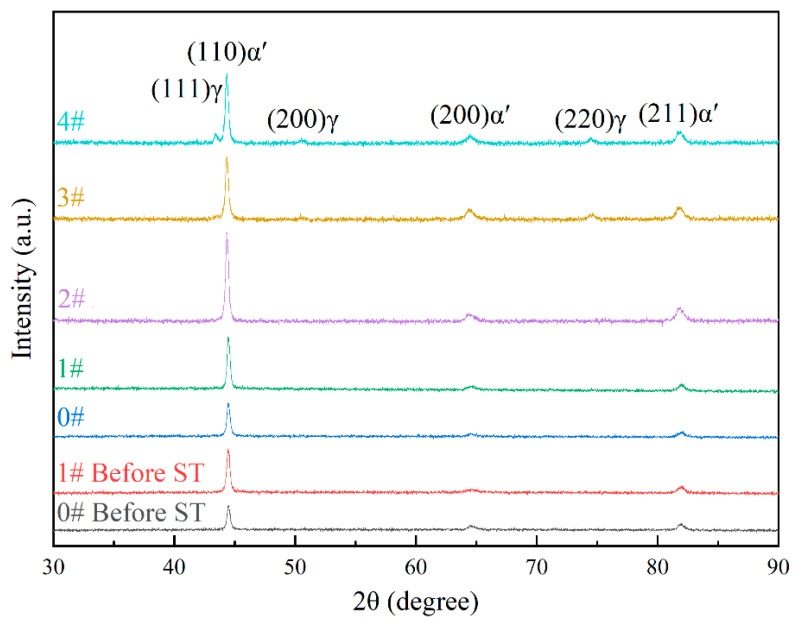
XRD patterns of experimental steel in different states.

To further determine the white granular structures in the microstructure and quantify their phase composition, X-ray diffraction (XRD) analysis was conducted on experimental steel in various states. As illustrated in Figure 6, the diffraction data were processed using Jade 6 software, revealing that both 0# and 1# specimens consist exclusively of martensite (α′) before and after solution treatment. The diffraction patterns are dominated by characteristic martensite peaks from the (110)α′, (200)α′, and (211)α′ crystal planes, with their corresponding 2θ angles at approximately 44°, 65°, and 82°, respectively. Cold deformation induces a notable increase in the intensity of the (110)α′ peak, accompanied by slight reductions in the (200)α′ and (211)α′ peak intensities. Significantly, the latter two peaks remain subdominant throughout, a trend consistently observed in the specimen after one time of solution treatment. Post-solution treatment, the (110)α′ peak intensity further increases relative to the as-deformed state, while the (200)α′ and (211)α′ peaks continue to show marginal intensity decreases.

Upon further comparison of the XRD phase analyses of specimens subjected to varying numbers of solution treatments, it is evident that both specimens 1# and 2# solely consist of the martensite phase. In contrast, when the number of solution treatments reaches 3 and 4, retained austenite emerges in the phase composition. A positive correlation is observed between the number of solution treatments and the intensity of the austenite diffraction peaks; specifically, as the number of solution treatments increases, the intensity of these peaks rises accordingly. In specimen 3#, the principal diffraction peak of austenite is identified as (220)γ, while the intensities of the (111)γ and (200)γ diffraction peaks are negligible. For specimen 4#, the dominant austenite diffraction peak shifts to (111)γ; the intensity of the (200)γ diffraction peak experiences a notable increase, whereas that of the (220)γ diffraction peak remains relatively stable. These findings strongly suggest that the white particles observed in the microstructure correspond to retained austenite. It should be noted that in certain specimens, the quantity of trace retained austenite is so minute (less than 1%) that it eludes detection during the XRD analysis.

To precisely quantify the relative fractions of the martensite and austenite phases, the diffraction peak intensities of each specimen were statistically analyzed via the Jade software. Subsequently, the relative phase fractions were calculated using the equations presented in Formulas (1) and (2) [18]:

(1) $$V_{\alpha^{\prime }}+V_{\gamma }=1$$

(2) $$V_{\gamma }=\frac{1.4I_{\gamma }}{I_{\alpha^{\prime }}+1.4I_{\gamma }}$$

In the above formulas, *V_α_*_′_ and *V_γ_* are employed to denote the volume fractions of martensite and austenite, respectively. Correspondingly, *I_α_*_′_ and *I_γ_* signify the diffraction peak intensities of martensite and austenite. Specifically, the intensities of their principal diffraction peaks are considered, and in this context, the areas of the diffraction peaks are utilized for analysis. For specimen 3#, the principal diffraction peaks are identified as (110)*α*′ and (220)*γ*. In contrast, for specimen 4#, the principal diffraction peaks are (110)*α*′ and (111)*γ*.

The outcomes of the analysis are presented in Table 3. No austenite diffraction peaks were discerned in specimens 1# and 2#, indicating a relative austenite content of 0%. Specimen 3# exhibited an austenite relative content of 4.5%, while specimen 4# had an austenite relative content of 8.9%.

In consideration of the potential occurrence of texture induced by cold deformation, the test result data were subjected to crystal plane texture analysis. The relative texture coefficients of crystal planes corresponding to each experimental steel were calculated by adopting Formula (3) [19]:

(3) $$RTc(hkl)=\frac{I_{\left(hkl\right)}/I_{0_{\left(hkl\right)}}}{\sum_{i=0}^{n}I_{\left(hkl\right)}/I_{0(hkl)}}$$

Within the formula, RTc(hkl) denotes the relative texture coefficient of the crystal plane. Specifically, $I_{\left(hkl\right)}$ represents the experimentally measured diffraction peak intensity of the specimen, while $I_{0_{\left(hkl\right)}}$ corresponds to the standard diffraction peak intensity documented in the JCPDS card. Additionally, *n* stands for the quantity of diffraction peaks.

Table 4 showcases the computational outcomes of the relative texture coefficients of crystal planes for the experimental steel across diverse states. Evidently, in all specimens, the crystal planes with the (110)α′ orientation hold the highest proportion. Cold deformation leads to a significant increase in the relative texture coefficient of the (110)α′ crystal plane, which rises from 37.5% in specimen 0# before aging treatment (0#-Before ST) to 52.0% in specimen 1# before aging treatment (1#-Before ST). Following one time of solution treatment, the relative texture coefficients of the (110)α′ crystal plane for specimens 0# and 1# ascend to 46.6% and 55.6%, respectively. This phenomenon will trigger a preferred orientation of the material along the (110)α′ crystal plane, consequently influencing the material’s practical application.

Nonetheless, as the number of solution treatments rises, the relative texture coefficient of the (110)α′—oriented crystal plane exhibits a declining tendency. Conversely, the relative texture coefficients of the (200)α′—and (211)α′—oriented crystal planes witness a corresponding increase. After three times of solution treatments, the relative texture coefficients of crystal planes in each orientation approach consistency, and the texture phenomenon gradually fades away.

### 3.2. Mechanism of Grain Refinement and Equiaxialization

Microstructural characterization (Figure 2, Figure 3 and Figure 4) demonstrates that cold deformation coupled with cyclic solution treatment effectively refines the original austenite grains and martensitic structure (statistical data are tabulated in Table 5). The optimal grain refinement is attained after three times of solution treatment subsequent to cold deformation, during which the grains gradually evolve toward an equiaxed morphology.

First, cold deformation induced fragmentation of the original austenite grains in the annealed experimental steel, accompanied by synchronous refinement of the internal martensite dimensions. Upon application of a specific deformation magnitude to the original austenite grains, a series of slip bands and high-density dislocations formed within the grains, progressively subdividing the original austenite into subgrains. With cumulative deformation, subgrain boundaries underwent gradual transformation into high-angle grain boundaries, thereby facilitating the formation of new grains and achieving mechanical fragmentation-induced refinement of the original austenite structure [10].

Based on the XRD characterization results, the Williamson-Hall method (i.e., Formulas (4) and (5)) was employed to quantify the dislocation density of the experimental steel under different processing states [20]:

(4) $$\beta cos\;\theta =\frac{K\lambda }{D}+4\varepsilon sin\;\theta$$

(5) $$\rho =\frac{14.4\varepsilon^{2}}{b^{2}}$$

In the formulas, *θ* denotes the angle corresponding to each diffraction peak, which must be converted to radians during calculations; *β* represents the full width at half maximum (FWHW) of each diffraction peak, similarly converted to radians for computation; *K* is the Scherrer constant, taking a value of 0.9 when *β* corresponds to the FWHW; *λ* is the X-ray wavelength, with Cu Kα_1_ set as 0.15406 nm; *D* signifies the grain size; *ε* is the microstrain; *ρ* represents the dislocation density; and *b* is the Burgers vector, specified as 0.249 nm for the martensite matrix. The *θ* and *β* values for each diffraction peak were derived by fitting the corresponding XRD data using Origin software. Substituting the *θ* and *β* values associated with the three diffraction peaks (110)α′, (200)α′, and (211)α′ of the specimen into Formula (4) generated a system of binary linear equations, from which the values of *D* and *ε* were solved. These parameters were subsequently substituted into Formula (5) to compute the *ρ* value. The dislocation density calculation results were tabulated in Table 6.

The results reveal that prior to solution treatment, cold deformation enhanced microstrain and dislocation density. Specifically, the dislocation density increased from 1.96 × 10^−3^ nm^−2^ in the specimen 0#-Before ST to 4.01 × 10^−3^ nm^−2^ in the specimen 1#-Before ST, representing a 104.6% increase. For the specimen 0# without cold deformation, the dislocation density after solution treatment (2.34 × 10^−3^ nm^−2^) exhibited a marginal increase compared to its pre-solution treatment state. This phenomenon arises because the absence of cold deformation resulted in insufficient dislocation density during the austenitization heating stage of the solution treatment process to satisfy recrystallization criteria. Consequently, during the cooling stage of the solution treatment process, the shear-type diffusionless phase transformation induced an elevation in dislocation density. In contrast, the dislocation density of specimen 1# decreased to below 1 × 10^−3^ nm^−2^ after solution treatment. Coupled with grain size statistics derived from microstructural morphology, it can be inferred that recrystallization occurred in the cold-deformed specimen 1# in the process of solution treatment.

During the heating stage of the solution treatment process, the austenite grain size is governed by two competitive processes: grain nucleation and growth. Cold deformation introduces an increase in dislocation density and high-angle grain boundaries, accompanied by irregular grain boundary morphology, which collectively provide abundant nucleation sites for austenitization. With prolonged heating, grain growth proceeds gradually. With high nucleation density facilitating rapid stabilization of grain boundary migration, at the austenitization start temperature (800 °C), complete transformation to austenitic structure is achieved. Upon further heating to the recrystallization temperature regime (800–900 °C), the inherited high-density dislocations and deformation-stored energy within grains create favorable thermodynamics for recrystallization nucleation, yielding new fine non-deformed grains that ultimately evolve into a uniform equiaxed microstructure [11].

In conclusion, cold deformation induces high-density dislocations and deformation-stored energy, which synergistically enhance austenitization nucleation efficiency and stabilization kinetics during the first solution treatment. Concurrently, these factors promote recrystallization, culminating in grain refinement and equiaxialization.

Secondly, cyclic solution treatment is capable of further refining the original austenite grains, consequently leading to the refinement of the martensite structure. During the water-cooling, a phase transformation from austenite to martensite takes place. The volumetric disparity between the two phases gives rise to the generation of dislocations. These dislocations are subsequently inherited in the subsequent solution treatment. When combined with the defects and deformation-stored energy, which were generated during the cold deformation process and remained uneliminated in the previous solution treatment, they continue to serve as the driving force for austenitization nucleation and recrystallization [21]. Consequently, the grains undergo further refinement.

Nevertheless, the grain-refining effect gradually diminishes with an increasing number of solution treatments. Evidently, this is because the quantity of dislocations generated by the phase transformation is significantly lower than that produced by cold deformation, which also highlights the significance of cold deformation in grain and structure refinement. The optimal grain refinement is attained after the third solution treatment. If the solution treatment is continued thereafter, the grains will not be refined; rather, they will experience a slight growth. This phenomenon primarily stems from the exhaustion of the defects and deformation-stored energy generated by cold deformation after multiple solution treatments, and the dislocations solely generated by the phase transformation are inadequate to supply the driving force necessary for austenitization nucleation and recrystallization.

### 3.3. Generation and Elimination of Grain Preferred Orientation

The relative texture coefficients of the crystal planes of the experimental steel under different states, as presented in Table 4, indicate that cold deformation induces the preferred orientation of crystals. This preferred orientation can be effectively eliminated through subsequent cyclic solution treatment. During cyclic solution treatment, re-austenitization and recrystallization take place, resulting in the consumption of micro-defects generated by cold deformation. In this process, new, non-deformed grains nucleate by consuming the deformation-stored energy. The equiaxed growth mechanism of these grains effectively eradicates the original crystallographic preferred orientation characteristics. As a fully recrystallized structure forms, the material ultimately exhibits an equiaxed grain structure with isotropic distribution, and the original deformation texture is entirely eliminated.

The XRD data corroborate the aforementioned view. As the number of solution treatments increases, the relative texture coefficient of the (110)α′ crystal plane of the tested steel diminishes, whereas the relative texture coefficients of the (200)α′ and (211)α′ crystal planes increase. The relative texture coefficients of these three planes gradually converge, which signifies the progressive completion of recrystallization and also denotes the gradual reduction and eventual disappearance of the texture within the structure.

The existence of cold deformation texture confers certain advantages to grain refinement during cyclic solution treatment [22,23,24,25]. During austenitization, the distinct atomic arrangements and inter-atomic spacings among different crystal plane families, along with the crystal orientation disparities induced by the texture, influence the dislocation density and distribution. This situation is favorable for austenite nucleation, with these regions acting as preferential nucleation sites. Furthermore, in materials exhibiting texture, the orientation constrains the migration of grain boundaries, impeding their movement and thereby suppressing grain growth.

The correlation between the data presented in Table 4 and Table 5 further validates this perspective. After undergoing cold deformation and three solution treatments, the 3# experimental steel exhibits the least pronounced texture. Concurrently, its grains and microstructure attain the highest level of refinement, which further attests to the rationality of the cold deformation—cyclic solution treatment process devised in this study.

### 3.4. Formation Mechanism of Retained Austenite

Cold deformation treatment exerts a non-negligible influence on the phase composition. XRD analysis reveals that the experimental steel before solution treatment consists exclusively of martensite phase; however, SEM observation indicates the presence of trace amounts of fine, white, dot-like retained austenite in the microstructure. Notably, the cold-deformed specimen 1#-Before ST exhibits a significantly lower density of these white dot-like features. As documented in Ref. [26], cold deformation triggers the transformation of retained austenite into martensite, a phenomenon associated with the transformation-induced plasticity (TRIP) effect. The underlying mechanism can be attributed to the inherently low stability of retained austenite, which requires a critical phase transformation driving force to convert into martensite. Cold deformation provides the necessary mechanical driving force to surmount this energy barrier, thereby promoting the transformation from retained austenite to martensite and consequently reducing the retained austenite content.

Upon multiple solution treatments of cold-deformed experimental steel, its phase composition transitions from a monophasic martensite structure to a biphasic mixture of martensite and retained austenite. Significantly, Table 3 demonstrates a positive correlation between the number of solution treatments and retained austenite content, with higher numbers of solution treatments corresponding to increased retained austenite retention.

The emergence of retained austenite is attributed to local Ni partitioning during cyclic solution treatments [27]. Diffusion processes, governed by chemical potential gradients, inherently proceed in the direction of decreasing chemical potential, where chemical potential is a function of both elemental concentration and temperature. Owing to the lower chemical potential of Ni in the austenite phase relative to martensite, Ni atoms migrate from martensitic domains to adjacent austenitic regions, creating Ni-enriched austenite and Ni-depleted martensite zones. This mechanism is corroborated by EDS point analysis (Table 2), which evidences slightly elevated Ni concentrations in retained austenite compared to martensite.

As a potent austenite-stabilizing element, Ni reduces the Ms temperature in a concentration-dependent manner. When local Ni enrichment surpasses a certain amount, the Ms temperature is depressed below room temperature, preventing transformation of Ni-rich austenite to martensite during water quenching. Consequently, these regions persist as retained austenite. This mechanistic framework explains both the appearance of retained austenite in the microstructure of maraging steel after cyclic solution treatments and the monotonic increase in retained austenite content with the number of solution treatments.

### 3.5. Mechanical Properties and Strengthening-Plasticizing Mechanism

#### 3.5.1. Mechanical Behavior

The microstructural evolution during aging of maraging steel is primarily characterized by the precipitation of alloy compounds and the formation of reverse transformation austenite, whereas the grain morphology and martensitic structure in the as-solutionized state remain essentially unaltered [28,29,30]. This highlights the critical need to investigate the mechanical properties and strengthening-plasticizing mechanisms of the solution-treated experimental steel for evaluating the final aged-state performance, with particular emphasis on plasticity. High-level plasticity in the solutionized condition serves as a foundational prerequisite for enhancing the overall ductility of maraging steel.

**Figure 7:**
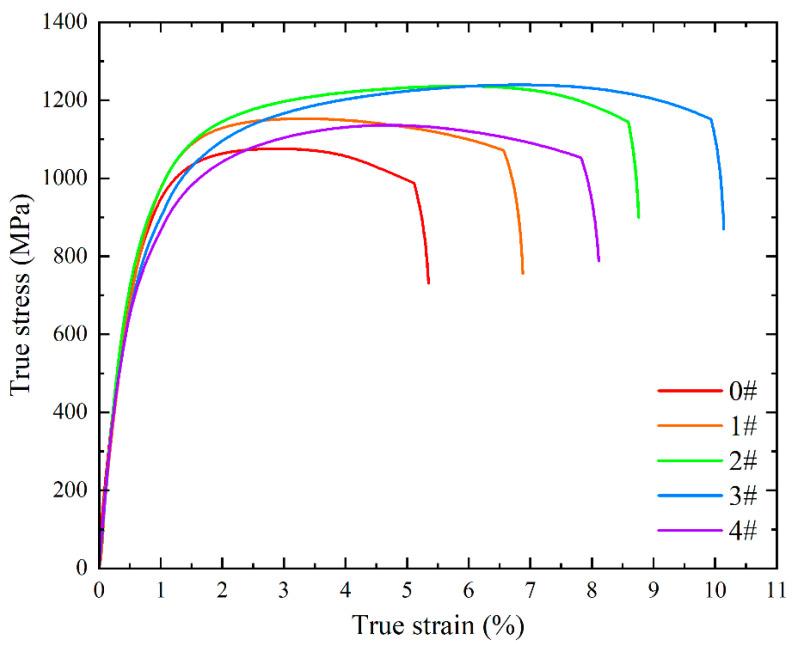
Room-temperature tensile true stress-strain curves of experimental steel in different states.

Room-temperature tensile testing was performed on the solution-treated specimens. Figure 7 illustrates the true stress-strain curves for each specimen, with corresponding mechanical property parameters tabulated in Table 7. Notably, cold deformation followed by solution treatment induced a significant enhancement in both strength and elongation. With increasing numbers of solution treatments, strength exhibited a non-monotonic trend, first increasing and then decreasing. The yield strength peaked at 881 MPa after two times of solution treatments, while the ultimate tensile strength reached a maximum of 1240 MPa after three times of solution treatments. Concurrently, elongation followed a similar trend, achieving a peak value of 9.93% after three times of solution treatments—nearly double the value of specimen 0#. Comprehensive performance evaluation indicates that the experimental steel subjected to cold deformation and three times of solution treatments displayed optimal room-temperature tensile properties.

#### 3.5.2. Strengthening Mechanism

In subsequent aging treatments, although changes in solid solution content, precipitated phases, and phase composition are induced, the original austenite grain structure and retained austenite phase remain fundamentally unaltered. Therefore, this paper focuses exclusively on the effects of these two microstructural features—original austenite grain size and retained austenite content—on the mechanical strength, with theoretical support from the fine-grain strengthening model (Formula (6)) [31]:

(6) $$\sigma_{gb}=\sigma_{0}+k_{y}d^{-1/2}$$

In the formula, $\sigma_{0}$ denotes the matrix yield strength, with a typical value of 50 MPa for iron-based materials; $k_{y}$ presents the slope of the $\sigma_{y}-d^{-1/2}$ re relationship, specified as 8.8 MPa·mm^1/2^ for 18Ni maraging steel [32]; and d corresponds to the average grain diameter. Converting martensite size to equivalent circular diameter for ease of calculation. The calculated fine-grain strengthening contributions for the experimental steels are 120 MPa, 148 MPa, 174 MPa, 201 MPa, and 193 MPa, respectively, as shown in Table 8. When compared with the corresponding tensile test strength data, these contributions exhibit a strong correlation with the strength increments. Cold deformation and cyclic solution treatment primarily affect the experimental steel by inducing grain refinement. Microstructural and mechanical property analyses reveal that as the number of solution treatments increases, the grain size first decreases substantially and then undergoes a slight increase, while the room-temperature tensile strength follows a corresponding trend of initial increase followed by decrease.

The strength of retained austenite is significantly lower than that of martensite, a characteristic that inherently tends to diminish the overall strength of the material. Notably, however, when compared to the 2# single-phase martensite specimen, the ultimate tensile strength of the specimen 3# did not decline with the introduction of austenite; instead, it reached a peak value of 1240 MPa. This phenomenon is attributed to the substantial strength contribution from grain refinement, which sufficiently counterbalanced the strength-degrading effect of the minor retained austenite phase. In the case of specimen 4#, where the retained austenite content reached 8.9%, the ultimate tensile strength decreased to 1136 MPa. Despite possessing finer grains and a more refined microstructure compared to the 1# and 2# specimens, the strength enhancement from grain refinement in the specimen 4# only marginally offset the detrimental impact of retained austenite. These results underscore that minimizing the content of retained austenite is critical for optimizing the strength of maraging steel, as the beneficial effects of grain refinement become insufficient to compensate for the strength loss once retained austenite exceeds a critical threshold.

#### 3.5.3. Plasticization Mechanism

**Figure 8:**
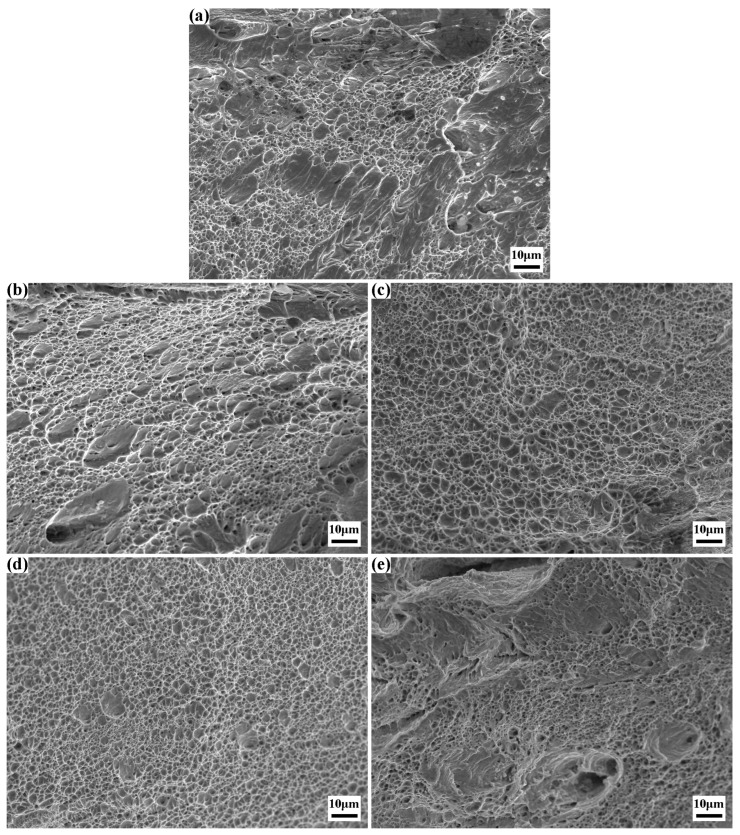
SEM morphologies of tensile fracture surfaces of experimental steel after solution treatment: (**a**) 0#; (**b**) 1#; (**c**) 2#; (**d**) 3#; (**e**) 4#.

The SEM fractographies of the tensile fracture surfaces for the experimental steel after solution treatment are presented in Figure 8. Specifically, Figure 8a illustrates a mixed fracture mode characterized by dominant ductile fracture with localized brittle fracture features. The surface displays dimples of non-uniform sizes and depths, with white tearing ridges distributed adjacent to both dimples and cleavage planes. The presence of dimples signifies that the specimen underwent substantial plastic deformation prior to fracture, whereby energy is dissipated through plastic deformation, thereby suppressing crack propagation.

Figure 8b exemplifies typical ductile fracture behavior, where dimples occupy nearly the entire fracture surface, and numerous submicron-scale microvoids are observed at the base of the dimples. This morphology indicates that fracture initiated and propagated via microvoid coalescence and growth mechanisms. With an increase in the number of solution treatments, all specimens’ tensile fracture surfaces predominantly exhibit ductile fracture characteristics. As shown in Figure 8c,d, the dimples are highly uniform, with the latter showing smaller dimple dimensions. Conversely, Figure 8e reveals a less regular fracture surface featuring larger zigzag voids and distinct brittle fracture zones, indicative of a mixed ductile-brittle fracture mode.

A comprehensive comparison reveals a remarkable correlation between the fracture morphology and the elongation of the specimens. For the specimen 0#, it features a relatively large proportion of dimple area and a small proportion of cleavage plane area. This distinct morphological characteristic is in excellent agreement with the 5.11% elongation obtained from the room-temperature tensile test. The specimen 1# demonstrates a notable plastic recovery phenomenon. As the proportion of dimples significantly increases and the cleavage planes are nearly absent, the specimen achieves an elongation of 6.57%. When it comes to the specimens that have undergone 2 and 3 times of solution treatment, their fracture morphologies are solely composed of dimples. In particular, the dimples in the specimen 3# treated with 3 times the solution treatment are more uniformly distributed compared to those in the specimen 2#. This more uniform dimple distribution is responsible for the optimal plasticity, with an elongation reaching 9.93%.

The microstructural features dictate both the fracture morphology and the plastic behavior of the material. First, grain refinement enhances the uniformity of plastic deformation and intergranular deformation coordination. Finer grains increase the grain boundary area, thereby elevating the probability of dislocation pile-up at boundaries. This mechanism promotes coordinated deformation between adjacent grains and homogenizes dislocation distribution within each grain, enabling more uniform plastic deformation throughout the material matrix. Second, uniformly equiaxed grain structures exhibit isotropic dimensional homogeneity, leading to balanced resistance against dislocation motion in all crystallographic directions during plastic deformation. This isotropy facilitates dislocation slip and multiplication, allowing the material to deform uniformly across all axes and mitigating anisotropy-induced local stress concentrations. Third, texture elimination removes restrictions on dislocation movement along specific crystallographic planes/directions, further enhancing deformation coordination and overall plasticity.

Notably, retained austenite—owing to its ductile nature—typically contributes positively to plasticity. In specimen 3#, the combined effects of 4.5% retained austenite and grain refinement synergistically maximize plasticity. However, in the specimen 4# subjected to four times of solution treatment, despite an 8.9% retained austenite content, grain coarsening dominates, causing elongation to decline. This underscores that the plastic benefit of minor retained austenite is subordinate to grain refinement, with the latter serving as the primary determinant of ductility.

## 4. Conclusions

Through SEM and XRD microstructural characterization and room-temperature tensile testing, the influence of cold deformation-cyclic solution treatment on the microstructure and mechanical properties of 18Ni(300) maraging steel prior to aging was systematically investigated. The key findings are summarized as follows:

(1) Compared with the traditional process, the cold deformation-cyclic solution treatment process can effectively refine the original austenite grains (minimum equivalent circle radius: 3.3 μm) and martensitic microstructure (minimum length and width: 7 μm and 1.3 μm, respectively) and render the original austenite grains uniformly equiaxed.
(2) Cold deformation introduces crystallographic preferred orientation (texture), which is mitigated through recrystallization during cyclic solution treatments. The interplay between texture generation and annihilation enhances grain refinement efficiency.
(3) Cyclic solution treatment promotes the formation of retained austenite within the martensitic matrix, with its volume fraction increasing monotonically with treatment cycles. After three cycles, the retained austenite content was 4.5%, and after four cycles, the retained austenite content was 8.9%.
(4) Grain refinement and uniform equiaxialization are the dominant strengthening-toughening mechanisms, contributing to concurrent improvements in strength and ductility. In contrast, retained austenite acts as a soft phase, reducing matrix strength while providing limited plasticity enhancement, necessitating strict compositional control.
(5) Under fixed solution parameters, the optimal number of solution treatments is deformation-dependent. For 30% cold deformation, three solution cycles at 860 °C for 10 min yield the most refined microstructure, fully equiaxed grains, complete texture elimination, and balanced retained austenite. This condition achieves the ideal strength-ductility balance (tensile strength of 1240 MPa and elongation of 9.93%), establishing a robust microstructural foundation for subsequent aging treatment.

## Figures and Tables

**Table 1:** Chemical compositions of the Co-saving 18Ni (300) test steel (wt.%).

Composition	Ni	Co	Mo	Ti	Al	Fe
Content	18.13	10.39	4.47	0.66	0.10	Bal.

**Table 2:** EDS point analysis results of the experimental steel in different states.

Specimen No.	EDS Point	Elemental Composition (wt.%)					
		Ni	Co	Mo	Ti	Al	Fe
0#-Before ST	Point 1 (Figure 3a)	18.72	10.12	5.08	0.62	0.15	Bal.
	Point 2 (Figure 3a)	18.07	10.30	5.85	0.55	0.19	Bal.
1#-Before ST	Point 3 (Figure 3b)	18.44	10.84	5.47	0.68	0.13	Bal.
0#	Point 1 (Figure 4a)	18.81	10.41	5.77	0.68	0.16	Bal.
	Point 2 (Figure 4a)	18.57	10.03	5.86	0.75	0.17	Bal.
1#	Point 1 (Figure 5a)	19.05	9.77	5.49	0.63	0.16	Bal.
2#	Point 2 (Figure 5b)	18.93	9.56	5.34	0.66	0.16	Bal.
3#	Point 3 (Figure 5c)	18.73	9.02	5.47	0.64	0.18	Bal.
	Point 4 (Figure 5c)	19.28	9.11	5.92	0.66	0.17	Bal.
4#	Point 5 (Figure 5d)	18.74	8.94	5.66	0.63	0.16	Bal.
	Point 6 (Figure 5d)	19.53	9.07	5.93	0.69	0.19	Bal.

**Table 3:** Relative phase contents of the tested steel after different times of solution treatment.

Specimen Number	Intensity of Principal Diffraction Peaks		Phase Content (%)	
	(110)α′	(111)γ/(220)γ	Martensite	Austenite
1#	28,088	0	100	0
2#	53,976	0	100	0
3#	25,866	874	95.5	4.5
4#	30,797	2144	91.1	8.9

**Table 4:** Relative texture coefficients of crystal planes of the experimental steel under different conditions (%).

Specimen Number	RTc(hkl)		
	(110)α′	(200)α′	(211)α′
0#-Before ST	37.5	29.8	32.7
1#-Before ST	52.0	20.1	27.9
0#	46.6	26.7	26.7
1#	55.6	20.1	24.3
2#	50.2	24.6	25.2
3#	40.8	32.4	26.8
4#	45.9	28.5	25.6

**Table 5:** Microstructural dimensions of the experimental steel under different conditions.

Specimen Number	Lath Martensite		Original Austenite Grain	
	Length (μm)	Width (μm)	Average Area (μm^2^)	Equivalent Circle Radius (μm)
0#	47	4.2	27,762	94
1#	18.3	2.8	317	10
2#	13.4	1.5	70	4.7
3#	7	1.3	34	3.3
4#	8.6	1.3	39	3.5

**Table 6:** Dislocation Density Calculation Results.

Specimen Number	(hkl)	θ (°)	β (°)	ε	ρ (nm^−2^)
0#-Before ST	(110)α′	44.50607	0.34703	2.89 × 10^−3^	1.96 × 10^−3^
	(200)α′	64.65564	0.97128		
	(211)α′	81.95592	0.62215		
1#-Before ST	(110)α′	44.48197	0.35606	4.14 × 10^−3^	4.01 × 10^−3^
	(200)α′	64.71126	1.66797		
	(211)α′	81.95428	0.67275		
0#	(110)α′	44.49842	0.36194	3.16 × 10^−3^	2.34 × 10^−3^
	(200)α′	64.60146	0.9415		
	(211)α′	81.98378	0.67046		
1#	(110)α′	44.50395	0.33175	1.94 × 10^−3^	0.88 × 10^−3^
	(200)α′	64.62762	0.84887		
	(211)α′	81.9837	0.53127		

**Table 7:** Room-temperature tensile mechanical property parameters of experimental steel in different states.

Specimen Number	Yield Strength (MPa)	Ultimate Tensile Strength (MPa)	Elongation (%)
0#	853	1076	5.11
1#	873	1153	6.57
2#	881	1236	8.59
3#	813	1240	9.93
4#	778	1136	7.82

**Table 8:** The fine-grain strengthening contributions of experimental steel in different states.

Specimen Number	Average Grain Diameter (μm)	The Fine-Grain Strengthening Contribution (MPa)
0#	15.86	120
1#	8.08	148
2#	5.06	174
3#	3.4	201
4#	3.77	193

## Data Availability

The original contributions presented in this study are included in the article. Further inquiries can be directed to the corresponding authors.
